# Dark adaptation in relation to choroidal thickness in healthy young subjects: a cross-sectional, observational study

**DOI:** 10.1186/s12886-016-0273-6

**Published:** 2016-07-11

**Authors:** Inger Christine Munch, Cigdem Altuntas, Xiao Qiang Li, Gregory R. Jackson, Oliver Niels Klefter, Michael Larsen

**Affiliations:** Department of Ophthalmology, Zealand University Hospital, Sygehusvej 10, DK-4000 Roskilde, Denmark; Faculty of Health and Medical Sciences, University of Copenhagen, Blegdamsvej 3, DK-2200 København N, Denmark; Department of Ophthalmology, Rigshospitalet, Nordre Ringvej 57, DK-2600 Glostrup, Denmark; MacuLogix, 1801 Oberlin Road, Middletown, PA 17057 USA

**Keywords:** Choroidal thickness, dark adaptation, time-to-rod-intercept, enhanced-depth optical coherence tomography, EDI-OCT

## Abstract

**Background:**

Dark adaptation is an energy-requiring process in the outer retina nourished by the profusely perfused choroid. We hypothesized that variations in choroidal thickness might affect the rate of dark adaptation.

**Method:**

Cross-sectional, observational study of 42 healthy university students (mean age 25 ± 2.0 years, 29 % men) who were examined using an abbreviated automated dark adaptometry protocol with a 2° diameter stimulus centered 5° above the point of fixation. The early, linear part of the rod-mediated dark adaptation curve was analyzed to extract the time required to reach a sensitivity of 5.0 × 10^−3^ cd/m2 (time to rod intercept) and the slope (rod adaptation rate). The choroid was imaged using enhanced-depth imaging spectral-domain optical coherence tomography (EDI-OCT).

**Results:**

The time to the rod intercept was 7.3 ± 0.94 (range 5.1 - 10.2) min. Choroidal thickness 2.5° above the fovea was 348 ± 104 (range 153–534) μm. There was no significant correlation between any of the two measures of rod-mediated dark adaptation and choroidal thickness (time to rod intercept versus choroidal thickness 0.072 (CI_95_ -0.23 to 0.38) min/100 μm, P = 0.64, adjusted for age and sex). There was no association between the time-to–rod-intercept or the dark adaptation rate and axial length, refraction, gender or age.

**Conclusion:**

Choroidal thickness, refraction and ocular axial length had no detectable effect on rod-mediated dark adaptation in healthy young subjects. Our results do not support that variations in dark adaptation can be attributed to variations in choroidal thickness.

**Electronic supplementary material:**

The online version of this article (doi:10.1186/s12886-016-0273-6) contains supplementary material, which is available to authorized users.

## Background

The choroid can be visualized in vivo by enhanced-depth imaging spectral-domain optical coherence tomography (EDI-OCT) [1]. Choroidal thickness has been measured in several studies and shown to vary nearly ten-fold among healthy individuals [2–5]. Choroidal thickness is associated with age [5, 6], gender [3], pubertal development [4], birth weight [7], and refraction and axial length of the eye with the choroidal thickness being thinner the longer and more myopic the eye [3–6]. The primary role of the choroid is believed to be that of supporting the function of the adjacent ocular structures given its rich network of vessels, the very high blood flow and the absence of a stroma [8]. The innermost layer of the choroid, the choriocapillaris, is of critical importance for retinal function as it nourishes the outer retina [8]. Its thickness ranges from 4–14 μm, decreasing with age according to a histological study of 95 healthy donor eyes from people between 6 and 100 years of age [9]. Thus, the extreme variation in the thickness of the total choroid lies in layers of Haller and Sattler, which have no known influence on retinal function.

Dark adaptation is an energy-requiring process in which the sensitivity of the retina to light increases a million-fold over 20–40 min [10]. The rate limiting step of this process is believed to be the recycling of retinoid to 11-cis retinal and the regenerating the visual pigment rhodopsin, which is an energy requiring process that takes place in the retinal pigment epithelium [10]. The process can be investigated by repeated testing of the light sensitivity threshold under scotopic conditions after having bleached the retinal photopigments [11]. The recovery of retinal sensitivity as a function of time after bleaching follows a biphasic curve when plotted on a logarithmic scale [11]. The first phase of the curve represents cone recovery. As the cone photoreceptors eventually reach a plateau of maximal sensitivity, the recovery of rod photoreceptor sensitivity can be detected [10]. The initial segment of both cone and rod recovery is exponential and then the rate of recovery decreases and reaches a plateau [10]. The reference method of measuring dark adaptation is Goldmann-Weekers dark adaptometry, that records full adaptation, including the phase of maximum rod sensitivity, which is reached in healthy subjects after approximately 40 min in darkness [11]. This method is relatively time-consuming for a clinical procedure and can be biased by patient fatigue [12]. An abbreviated method has therefore been developed [12]. It measures the rate of dark adaptation from bleaching to a standard sensitivity threshold rather than following dark adaptation to the final rod threshold. Its design gives priority to brevity of examination and therefore uses a weaker bleaching flash than the Goldmann-Weekers adaptometer. Consequently, the shape of the cone phase of the adaptation curve is less distinctly defined with the new instrument [13]. If cone adaptation is fast, a linear recovery may be exhibited [13]. If the patient has impairment of cone adaptation, a full exponential recovery may be observed [14].

We have examined the effect of choroidal thickness on retinal function using such an abbreviated dark adaptometry protocol to measure the rate of rod dark adaptation in healthy young adults.

## Methods

This study was appended to a study of choroidal thickness in young adults [3], who were recruited by posting a call on the internal website for medical students of the Faculty of Health Sciences of the University of Copenhagen. Exclusion criteria included previous eye trauma or ocular surgery, congenital malformations of the eye, amblyopia, and inability to cooperate. Forty-three of the 93 participants in the core study volunteered to participate in the ancillary study and underwent dark adaptometry in a separate session. One participant was excluded because of inability to perform dark adaptometry, thus leaving 42 subjects for analysis. Examinations took place from April 2010 to June 2010 and from January 2011 to February 2011. The study was conducted in accordance with the ethical standards stated in the Declaration of Helsinki and approved by the local medical ethics committee, ‘De Videnskabsetiske Komiteer for Region Hovedstaden’. Written informed consent was obtained from all participants.

Original study parameters and procedures included past medical history, current medications, non-cycloplegic and cycloplegic objective refraction, best-corrected visual acuity, arterial blood pressure manometry, ocular axial length and digital fundus photography. Enhanced-depth imaging optical coherence tomography (EDI-OCT, Spectralis, Heidelberg Engineering, Heidelberg, Germany) was made in the form of seven parallel line scans covering a 5° vertical by 30° horizontal fovea-centered area of the fundus with real-time averaging of 25 B-scans per line. The line closest to the foveal center was identified by the depth of the foveal depression. Subfoveal choroidal thickness was measured by a single observer who manually moved the segmentation line, which was first placed automatically at the inner limiting membrane, to the choroidoscleral border using the instrument manufacturer’s proprietary software (Heidelberg Eye Explorer version 1.6.1.0). The segmentation line placed automatically on Bruch’s membrane was checked for accuracy and was kept unchanged in all subjects. Comparison of interobserver variability in 15 subjects in the core study showed no significant effect of observer [3]. For the present study, choroidal thickness was measured at the center of the most superior of the seven line scans, which was closest to the area investigated by the dark adaptometer.

Dark adaptometry was performed as described by Jackson and Edwards [12] using a computer-controlled instrument (AdaptDx, MacuLogix, Hummelstown, PA, USA) in the study eye after pupil dilation to a diameter of ≥6 mm using topical phenylephrine hydrochloride 10 % (Optha A/S, c/o Actavis Nordic, Denmark) and tropicamide 1 % (Alcon, Denmark) instilled no less than 15 min before the examinations. The fellow eye was occluded with an eye patch. A built-in infrared camera continuously monitored the test eye. The operator centered the subject’s test eye on a red fixation light with the help of a reticule overlaid on a computer display of the test eye. A trial lens matched to the subject’s objective refraction was placed in front of the subject’s eye as needed. All subjects trained the task at both high and low light intensities to become familiar with the procedure before beginning the examination.

The test eye was bleached by exposure to a white photoflash (0.25 ms duration, 6.38 log scotopic Trolands-second) while the subject focused on the fixation light. A stimulus of 500 nm wavelength with a 2° diameter was centered 5° below the point of fixation. Starting at a high intensity (5.00 cd/m^2^), stimuli were presented every 2 or 3 s for a duration of 200 ms. If the subject did not respond by manually pressing a button within 2 s of the stimulus onset, the stimulus intensity remained unchanged on successive stimulus presentations until the subject responded. If the subject responded to the stimulus, the intensity was decreased for each successive presentation in steps of 0.3 log units until the subject stopped responding. When the subject stopped responding, the intensity was increased 0.1 log units for each successive presentation until the subject responded again. This intensity was taken to represent the threshold for a given interval after bleaching. Subsequent threshold assessments started with a stimulus intensity 0.2 log units brighter than the previous threshold measurement. The subject had a rest period of 10 s between threshold measurements.

**Fig. 1:**
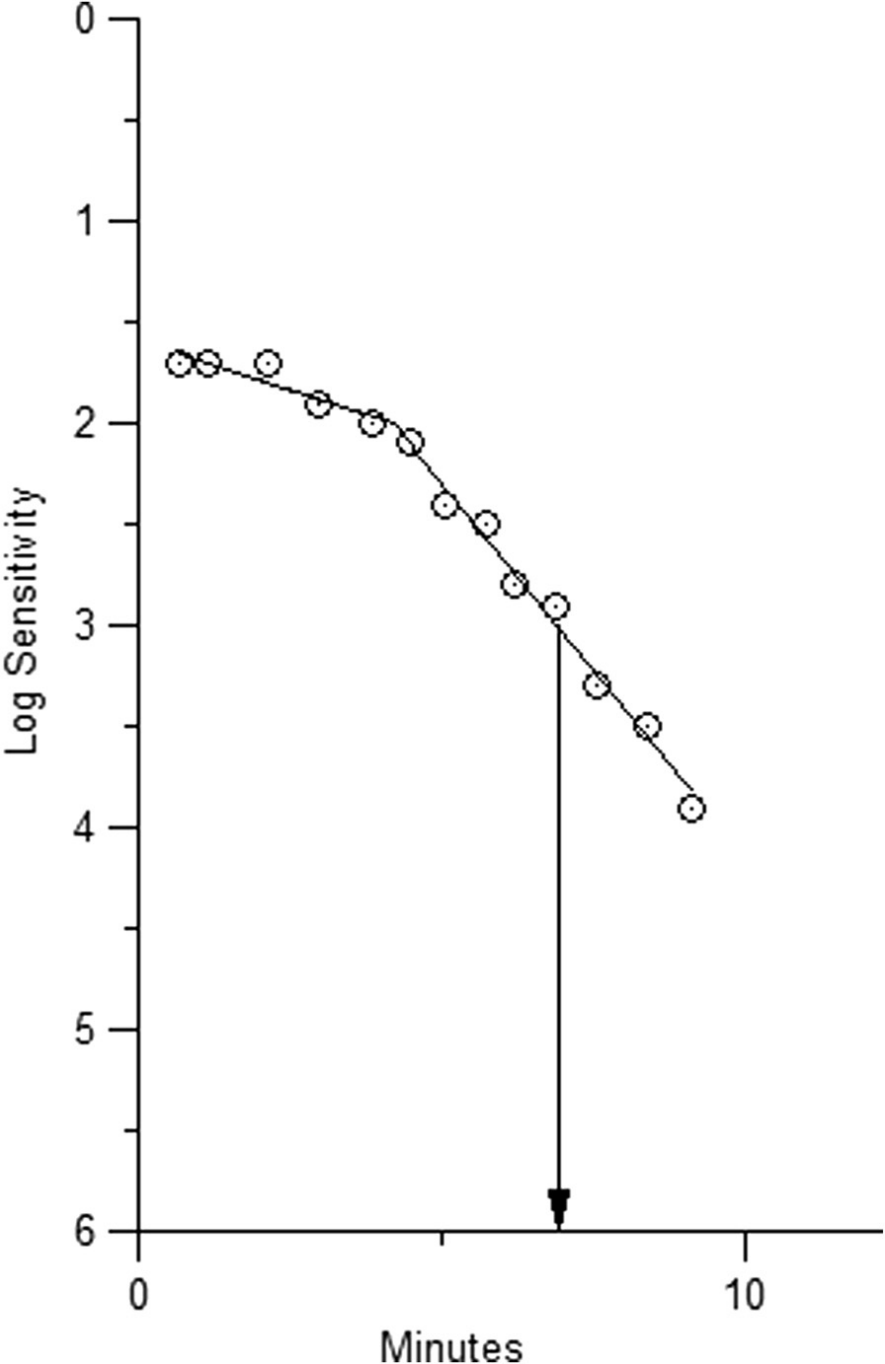
Dark adaptation curve from a 25-year-old female participant with a choroidal thickness of 310 μm. At time 0 the retina was partially bleached with a white photoflash (0.25 ms duration, 6.38 log scotopic Trolands-second). The dark adaptation curve does not exhibit the initial exponential part of the cone adaptation as the cone adaptation was fast and the retina not fully bleached. The recovery time to reach the pre-specified sensitivity threshold of 3 log units was 6.9 min. The rod adaptation rate was 0.37 log units/min as estimated from the slope of the second part of the curve, which represents the rod-mediated dark adaptation

Dark adaptation curves were modeled using the SAS software package (version 9.1, SAS Institute, Cary, NC, USA). A single-exponential, single-linear model was used as previously described [15] with the initial exponential part of the curve corresponding to cone-mediated dark adaptation and the linear part of the curve corresponding to the initial part of rod-mediated dark adaptation (Fig. 1). The rod adaptation rate was estimated by the slope of the linear part of the model. The rod intercept was calculated as the intercept between the linear part of the dark adaptation curve and the pre-defined sensitivity threshold of 5.00 × 10^−3^ cd/m^2^ corresponding to 3.0 log units. The adaptometer was set to terminate the test after 20 min, or sooner, if the predefined threshold had been reached. Consequently, the test was not designed to determine final rod threshold. All measurements were performed during daytime between 9am to 4pm.

Statistical analyses were made using the SAS software package 9.1. Mean and standard deviation or median and interquartile range were calculated for continuous variables and compared between genders using unpaired, two-tailed Student’s t-tests (choroidal thickness, axial length, blood pressure, age, time to rod intercept and rod adaptation rate) or two-tailed Mann Whitney rank sum test (refraction) as the distribution of the spherical equivalent was skewed. A general linear model (PROC GLM) was used to describe associations between the measures of dark adaptation (time to rod intercept, rod adaptation rate) and choroidal thickness, axial length, refractive error, blood pressure, age and gender. Linear regression models were tested for linearity, variance homogeneity and normality of the distribution of residuals by visual inspection of relevant plots. The correlation between subfoveal choroidal thickness and time to rod intercept was adjusted for age and sex by including age as a continuous variable and sex as a class variable in the linear regression model. The level of statistical significance was set to P < 0.05 and estimates presented with 95 % confidence intervals (CI_95_). Power estimation was performed using the analyst application in SAS. The spherical equivalent refraction error was used in analysis and calculated as the algebraic sum of the value of the sphere and half the cylindrical value.

## Results

Dark adaptometry data were available in 42 subjects (12 men and 30 women, 31 right eyes and 11 left eyes) with a mean age of 25.0 years (range 21–30 years) (Table 1). All eyes had a best corrected visual acuity of Snellen 0.8 or better and 39 eyes had 1.0 or better. Median spherical equivalent refraction was −0.56 D ± 2.5 (interquartile range, IRQ) (range −11.3D to +3.63D) (Table 1). The mean axial length was 24.0 mm ± 1.1 (range 21.2–27.3mm). Choroidal thickness at 2.5° above the fovea was normally distributed with a mean of 348 μm ± 104 (range 153–534 μm) (Table 1). None of the participants had a history of retinal or uveal disease and the posterior pole was unremarkable in all participants.

**Table 1:** Characteristics of the study population of 42 healthy university students

	All subjects	Men	Women	*P*-Value*
Number	42	12 (29%)	30 (71%)	
Age, years	25.0 ± 2.0 (21–30)	25.3 ± 2.2	24.8 ± 1.9	0.47
Systolic blood pressure, mmHg	124 ± 13.5 (95 to 154)	132 ± 15.4	121 ± 11.2	0.0094
Diastolic blood pressure, mmHg	77 ± 11.5 (52–104)	77 ± 12.2	78 ± 11.4	0.59
Choroidal thickness, μm	348 ± 104 (153–534)	380 ± 93	336 ± 107	0.22
Axial length, mm	24.0 ± 1.1 (21.2–27.3)	24.1 ± 0.87	24.0 ± 1.3	0.67
Spherical equivalent refraction, D	−0.56 ± 2.5 (−11.3 to 3.63)	0.0 ± 0.81	−1.25 ± 4.0	0.14
Time to rod intercept, min	7.3 ± 0.94 (5.1–10)	7.3 ± 1.3	7.4 ± 0.80	0.88
Rod adaptation rate, log /min	0.34 ± 0.055 (0.22–0.46)	0.36 ± 0.058	0.33 ± 0.053	0.098

Data presented as mean ± standard deviation (range) or fraction (%) except refraction that is presented as median (interquartile range) because of a skewed distribution

Choroidal thickness was measured 2.5° above the fovea

* Two-tailed Student’s unpaired *t*-test or in case of refraction two-tailed Mann Whitney rank sum test

**Fig. 2:**
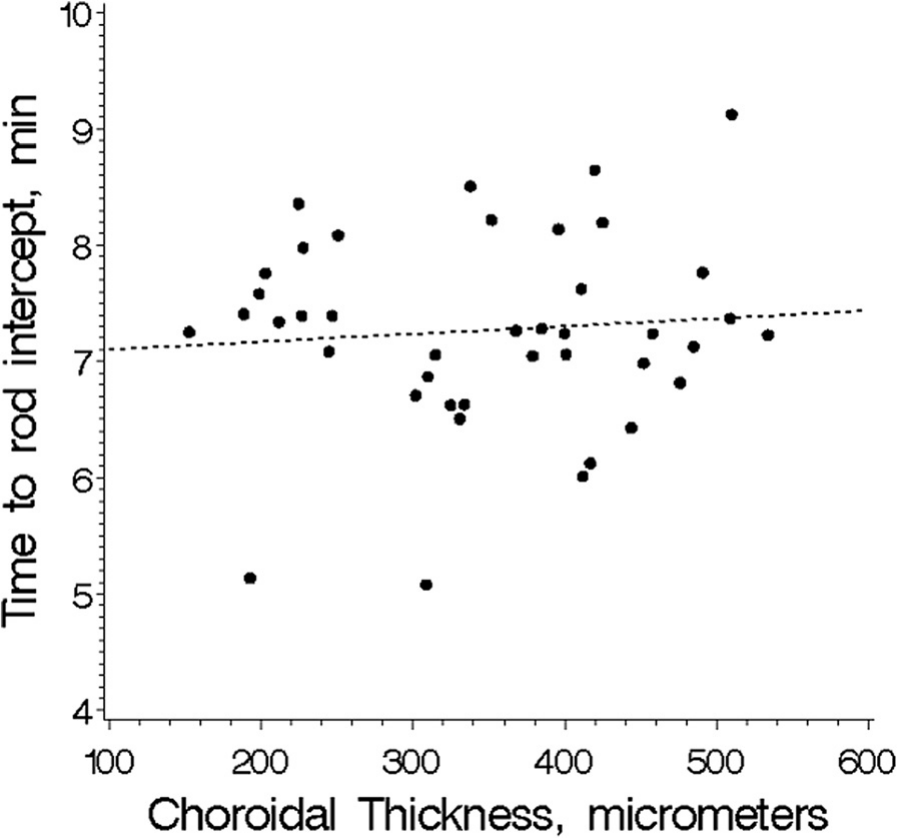
Dark adaptation in relation to choroidal thickness in 42 healthy, young subjects. The speed of dark adaptation was measured as the time to reach a predefined sensitivity threshold (the rod intercept). The slope of the regression line is 0.076 (CI_95_−0.21 to 0.37) min/100μm (P = 0.64, adjusted for age and sex)

Time to rod intercept was normally distributed with a mean of 7.3 min ± 0.94 (range 5.1–10 min) and comparable between men and women (Table 1). An example of a dark adaptation curve from one of the participants is provided in Fig. 1. There was no significant correlation between time to rod intercept or rod adaptation rate and choroidal thickness with the linear regression coefficient for time to rod intercept being 0.072 (CI_95_−0.21 to 0.37, P = 0.64) min per 100 μm increase in choroidal thickness, adjusted for age and sex (Fig. 2, Table 2). When the choroidal thickness was included as a dichotomous variable (choroidal thickness greater or smaller than the median of 359 μm) the rod intercept was numerically 0.29 min (CI_95_ 0.25–0.30 min) longer in the eyes with the thicker choroids (P = 0.57 adjusted for age and sex) (data not tabulated). There was no significant association between the rod-mediated dark adaptation (time to rod intercept, rod adaptation rate) and age, sex, refractive error, axial length or blood pressure (Table 2). Including refraction, axial length and the blood pressure in the analyses of the correlation of time to rod intercept and choroidal thickness did not alter the results (data not tabulated).

**Table 2:** Correlations of rod-mediated dark adaptation in 42 healthy subjects

	Time to rod intercept (CI_95_)	P	Rod adaptation rate (CI_95_)	P
Choroidal Thickness	0.072 (−0.23 to 0.38) min/100μm	0.64	−0.0035 (−0.021 to 0.014) log10/min/100μm	0.68
Axial Length	−0.12(−0.38 to 0.15) min/mm	0.39	−0.00035 (−0.016 to 0.015) log10/min/mm	0.96
Refraction	0.0087 (−0.13 to 0.11) min/D	0.88	0.0011 (−0.0078 to 0.0055) log10/min/D	0.74
Age	−0.051 (−0.21 to 0.10) min/year	0.50	0.0014 (−0.0073 to 0.010) log10/min/year	0.74
Sex (women compared with men)	0.025 (−0.64 to 0.69) min	0.94	−0.031 (−0.069 to 0.0075) log10/min	0.11
Systolic blood pressure	−0.16 (−0.41 to 0.08) min/10mmHg	0.19	−0.0031 (−0.017 to 0.011) log10/min/10mmHg	0.66
Diastolic blood pressure	−0.089 (−0.36 to 0.19) min/10mmHg	0.52	−0.0045 (−0.020 to 0.011) log10/min/10mmHg	0.57

Table values refer to linear regression coefficients and are adjusted for age and sex by including age and sex in the statistical model

The study had a power of 95 % to detect a response parameter difference of 15 % between the one half of participants with the thicker choroids compared with the other half with the thinner choroids at a significance level of 0.05. The raw data for this study are available as Additional file 1.

## Discussion

This study did not detect any correlation between choroidal thickness and rod-mediated dark adaptation as measured by time to rod intercept and rod adaptation rate in healthy, young medical students.

Dark adaptation is an energy-requiring process that is sensitive to oxygen tension [16], and glycemia [15], suggesting that it might also be sensitive to choroidal thickness as total choroidal thickness has been shown to be correlated with choroidal blood flow [17] and may thus influence the retinal supply of glucose and oxygen. We found no such effect, which supports - within the limitations of the study – that the rate of dark adaptation is not dependent on the total thickness of the choroid in healthy eyes. We also showed that the dark adaptation did not depend on refraction, axial length of the eye or on blood pressure in this group of young adults. Apparently, the choriocapillaris alone, with its high blood flow [8] and the low extraction of oxygen from the choroidal blood, makes the rod photoreceptors independent on the outer layers of the choroid [18].

The AdaptDx adaptometer is supplied with a short-duration dark adaptation protocol for detection of age-related maculopathy [12]. Less intense pre-adaptation bleaching compared with classic adaptometry [19] shortens the time to rod recovery. The final rod threshold is not measured but the time to reach a predefined threshold and the slope of the first, linear part of the rod adaptation curve are calculated. The test stimulus is placed 5° above the fovea rather than 12° from the fovea where the rod density peaks [20], as is used in traditional dark adaptometry [19]. This reflects the priority given to age-related macular degeneration.

In theory, the rod adaptation rate is a relatively robust measure of rod adaptation compared with full threshold measurements because it is independent of magnification effects and pupil size and the risk of patient fatigue is reduced by the shorter duration of the test [12].

Limitations of the study included the lack of assessment of the final rod threshold. Hence, we cannot rule out the existence of an association between the final rod threshold and choroidal thickness. The choroidal thickness was measured 2.5° above the fovea and not 5° above the fovea where the stimulus for dark adaptometry was placed. It is, however, unlikely that is would have affected the results as a previous study did not find any significant difference in macular choroidal thickness along the vertical meridian through the fovea [5]. The choroidal thickness was not measured in conjunction with the dark adaptometry but several months before. The power of the study to detect minor associations was limited by the number of subjects included. Furthermore, the results are limited to healthy young subjects and does not include information about acute changes in choroidal thickness during dark adaptation, if present.

Choroidal thickness in this study was comparable with other studies of young adults [5] or children [4]. Choroidal thickness has been reported to be thinner in middle-aged and elderly subjects than in the young [6, 21], in agreement with a thinner choroidal thickness being associated with higher age in cross-sectional studies [5, 6, 21, 22].

The mean time to rod intercept and the slope of the linear part of the rod-mediated dark adaptation measured in this group of young healthy adults were comparable with previous studies [12]. In patients with diabetes and minimal retinopathy, the time to rod intercept has been reported to be longer and the adaptation rate slower [15, 23]. In subjects with age-related macular degeneration the dark adaptation rate is severely prolonged [12, 13].

Myopia is strongly associated with a longer axial length and a thinner choroid [3–6]. Previous studies have found impaired macular sensitivity and impaired dark adaptation in relation to high myopia [24–26]. The rate of dark adaptation decreases with age [27] as does choroidal thickness [5, 6, 21]. The effect of choroidal thickness on dark adaptation has not previously been investigated. The present study suggests that the effects of refraction and age are not mediated by variations in choroidal thickness within the range we investigated (153–534μm).

Our study measured rod function because it is highly sensitive to oxygen and because our primary suspicion was that because gasses diffuse easily, a thicker choroid might provide a higher oxygen delivery to the retina. Higher oxygen delivery was thought to provide faster dark adaptation because dark adaptation has been found to be accompanied by a 50 % increase in the oxygen consumption of the outer retina in cats [28]. There is, to the best of our knowledge, no information available about the relation between total choroidal thickness and choriocapillaris perfusion or the contribution of the outer layers of the choroid to the supply of oxygen and glucose to the retina. The present study does not add any direct information about this topic, but it is compatible with the thickness of the outer choroid having no influence on retinal function.

The Beijing Eye Study found that extremely thin subfoveal choroidal thickness (<30μm) was associated with lower visual acuity in a multivariate analysis after excluding eyes with disease [29]. It seems plausible that the choriocapillaris is disturbed only in eyes with an extremely thin choroid. Our study did not include participants with choroidal thickness less than 153 μm. Hence, we have no data on the association between dark adaptation and having an extremely thin choroid.

## Conclusion

In conclusion, we did not find any correlation between the rate of rod dark adaptation and choroidal thickness in healthy young subjects. We found no evidence that variations in dark adaptation can be attributed to variations in choroidal thickness.

## Additional file

Additional file 1: Raw data, containing the raw data sheet that was used for the statistical analyses. (XLS 32 kb)
